# Identification and Characterization of a New Enterotoxin Produced by *Clostridium perfringens* Isolated from Food Poisoning Outbreaks

**DOI:** 10.1371/journal.pone.0138183

**Published:** 2015-11-19

**Authors:** Daisuke Irikura, Chie Monma, Yasunori Suzuki, Akiko Nakama, Akemi Kai, Aya Fukui-Miyazaki, Yasuhiko Horiguchi, Tomoya Yoshinari, Yoshiko Sugita-Konishi, Yoichi Kamata

**Affiliations:** 1 Division of Microbiology, National Institute of Health Sciences, Tokyo, Japan; 2 Department of Microbiology, Tokyo Metropolitan Institute of Public Health, Tokyo, Japan; 3 Department of Molecular Biology, Research Institute of Microbial Diseases, Osaka University, Osaka, Japan; 4 Department of Food and Life Sciences, The Graduate School of Life and Environmental Sciences, Azabu University, Kanagawa, Japan; 5 Department of Veterinary Medicine, Iwate University, Morioka, Japan; Institute Pasteur, FRANCE

## Abstract

There is a strain of *Clostridium perfringens*, W5052, which does not produce a known enterotoxin. We herein report that the strain W5052 expressed a homologue of the iota-like toxin components sa and sb of *C*. *spiroforme*, named *Clostridium perfringens* iota-like enterotoxin, CPILE-a and CPILE-b, respectively, based on the results of a genome sequencing analysis and a systematic protein screening. In the nicotinamide glyco-hydrolase (NADase) assay the hydrolysis activity was dose-dependently increased by the concentration of rCPILE-a, as judged by the mass spectrometry analysis. In addition, the actin monomer of the lysates of Vero and L929 cells were radiolabeled in the presence of ^[32P]^NAD and rCPILE-a. These findings indicated that CPILE-a possesses ADP-ribosylation activity. The culture supernatant of W5052 facilitated the rounding and killing of Vero and L929 cells, but the rCPILE-a or a non-proteolyzed rCPILE-b did not. However, a trypsin-treated rCPILE-b did. Moreover, a mixture of rCPILE-a and the trypsin-treated rCPILE-b enhanced the cell rounding and killing activities, compared with that induced by the trypsin-treated rCPILE-b alone. The injection of the mixture of rCPILE-a and the trypsin-treated rCPILE-b into an ileum loop of rabbits evoked the swelling of the loop and accumulation of the fluid dose-dependently, suggesting that CPILE possesses enterotoxic activity. The evidence presented in this communication will facilitate the epidemiological, etiological, and toxicological studies of *C*. *perfringens* food poisoning, and also stimulate studies on the transfer of the toxins’ gene(s) among the Genus *Clostridium*.

## Introduction

*Clostridium perfringens* is a toxin-producing bacterium, causing gas gangrene and food-borne illnesses in human and digestive diseases in other animals. *C*. *perfringens* produces four typing toxins (alpha, beta, epsilon, and iota) and at least eleven other toxins [1–3]. *C*. *perfringens* is classified into five types, A through E, on the basis of its production of the four typing toxins. Enterotoxin (CPE) is one of the toxins produced by *C*. *perfringens*, evoking diarrhea and the enterotoxin gene is cloned and sequenced [4]. Most CPE-producing *C*. *perfringens* belong to type A. Type E *C*. *perfringens* produces alpha and iota toxins and leads to antibiotic-associated enterotoxaemia in rabbits and sporadic outbreaks in bovine and ovine species [5]. *C*. *spiroforme* causes enteritis in rabbits [6]. The production of an iota toxin homologue, iota-like toxin, by *C*. *spiroforme* was reported previously [7]. Our survey of the literature found no reports indicating that type E *C*. *perfringens* causes any diseases in humans.

Type E *C*. *perfringens* specifically produces iota toxin. Iota toxin consists of two components; enzymatic and binding components, named ia and ib, respectively. Iota toxin is a member of the binary toxin group [8], which also includes *C*. *botulinum* C2 toxin (C2I and C2II)[9] and *C*. *difficile* ADP-ribosyltranferase (cdtA and cdtB) [10]. Iota-like toxin produced by *C*. *spiroforme* also belongs to the binary toxin group. Components of iota-like toxin are designated as Sa and Sb [11]. Iota toxin ib is produced as inactive precursors. An N-terminal region is then removed by bacterial proteases after secretion from the bacterial cell body, and then both components become active [12]. Iota toxin ia mediates ADP-ribosylation by catalyzing the nicotinamide glyco-hydrolase (NADase) reaction and the transfer of the ADP-ribose to intracellular actin monomers. Component ia is known to contain three conserved regions: the arginine residue as the catalytic center for both reactions, a Glu-X-Glu (EXE) motif, and an STS motif. The EXE motif, located in the ADP-ribosylating tune-tune loop, is particularly important for the enzymatic activity [13–15]. Iota toxin ib displays significant homology to the protective antigen of anthrax toxin (54.5% similarity overall) and C2II (39.0% similarity overall) [16].

The molecule of iota toxin ib and its homologues are divided into four domains. Each domain possesses distinct functions, such as binding to the cells, oligomerization of the binding components, insertion of the binding components into the membrane, and binding to the enzymatic component ia [17]. Recently, iota toxin ib was found to mediate the internalization of ia into the cytosol [18]. After transfer of ADP-ribose to globular actin by ia, depolymerization of the actin cytoskeleton occurred, and then cell rounding and cell death were evoked in various mammalian cell lines including L929 cells not Vero cells [19].

It is necessary to determine the presence of the *cpe* gene and the production of CPE protein in isolates from the affected patients/foods in order to diagnose *C*. *perfringens* type A food poisoning. PCR and the reversed-passive latex agglutination test are available for the detection of the *cpe* gene and CPE protein. In 1997, we encountered a strange outbreak of food poisoning in Japan. Although the clinical symptoms of the patients and epidemiological characteristics indicated that the outbreak was caused by *C*. *perfringens*, the isolates of *C*. *perfringens* did not harbor the *cpe* gene nor produce CPE protein in culture. Three more outbreaks (for a total of two in Tokyo, one in Osaka, and one in Tochigi) were identified [20]. The culture supernatant of the *C*. *perfringens* strain W5052 originated from the outbreak that occurred in Tokyo in 1997 evoked the death of the L929 and Vero cells. The supernatant also evoked swelling and fluid accumulation of the ileal loops of rabbits, suggesting the presence of enterotoxin. It is well known that CPE kills Vero cells, but not L929 cells, because the L929 cells do not harbor the receptor for CPE [21]. Anti-alpha toxin and anti-CPE antibodies did not neutralize the cell killing and enterotoxic activity of the strain W5052 culture supernatant. These findings suggested that the strain W5052 produces an unidentified enterotoxin.

At the International Conference on the Molecular Biology and Pathogenesis of Clostridia-ClostPath 2013, we presented the evidence for a new, previously unknown enterotoxin. We named the toxin as *Clostridium perfringen*s iota-like enterotoxin, CPILE, on the basis of a genomic DNA sequence analysis of the causative *C*. *perfringens* strain W5052 using next generation DNA sequencing and systematic protein screening by mass spectrometry. In this communication, we present the processes by which CPILE was screened and identified, and the properties of CPILE as characterized by the molecular biological, protein-chemical, and cellular biological methods. The information revealed here promises to improve the epidemiological study of *C*. *perfringens* food poisoning and research into the toxins produced by the Genus *Clostridium*.

## Materials and Methods

### Strain and culture

*C*. *perfringens* strain W5052, which lacks *cpe* gene and the expression of CPE, was isolated from the diarrheal feces of patients during a food poisoning outbreak that occurred in Tokyo in 1997 [20]. Three similar outbreaks occurred in 2003 in Tokyo, 2009 in Osaka, and 2010 in Tochigi, all lacking CPE, were also used. Strain W5052 was maintained in a cooked meat medium (CMM, BD). The suspended culture supernatant of the stocked CMM was inoculated into a new CMM and cultured overnight at 37°C. A tiny volume of the suspension of the culture supernatant was inoculated in a Brain Heat Infusion (BHI) medium (BD) and was cultured overnight at 37°C. A 1% of the supernatant cultured in BHI medium was inoculated into a modified Duncan-Strong (DS) medium [22], and cultured for four days at 37°C for identification of the production and for purification of the new enterotoxin.

### Genomic sequencing and bioinformatic studies of *C*. *perfringens* strain W5052

The genomic DNA of the strain W5052 was purified from the BHI culture medium using a DNeasy Blood & Tissue Kit (Qiagen). The sequence of the genomic DNA was determined by a Roche Genome Sequencer FLX. This analysis was carried out by Hokkaido System Science Inc. (Hokkaido, Japan). Jemboss, a computer software program, was used to predict possible open reading frames [23]. Homologues to the deduced amino acid sequence were searched using the BLAST program [24]. A multiple sequence alignment was performed using the Genetyx software program (Software Development Inc., Tokyo, Japan).

#### Partial purification of a new enterotoxin produced by *C*. *perfringens* strain W5052

The strain W5052 was cultured in modified DS medium. After centrifugation of the culture at 10,000 x g for 10 min at 4°C, the clear supernatant was harvested. Ammonium sulfate was added into the culture supernatant to achieve 0–70, 0–40, 40–50, 50–60, and 60–70% saturation fractions. The protein precipitates were harvested by centrifugation at 10,000 x g at 4°C for 10 min, suspended in a small volume of phosphate-buffered saline (PBS), and then dialyzed against PBS. The suspended precipitates were cleared by filtration with a 0.2-μm filter (Techno Plastic Products). The protein concentration of the fractions was determined using a BCA protein assay kit (Pierce, Rockford, IL USA). The final products were kept at -80°C until protein mass spectrometry analysis and determination of cell toxicity.

A fraction containing cytotoxic activity was subjected to FPLC-gel filtration on a Superdex 200 10/300 GL column (GE Healthcare) using an AKTA Purifier. The solvent was PBS, with a flow rate of 0.5 ml/min.

### Mass spectrometry to identify the proteins

A fraction containing cytotoxic activity was digested by trypsin after reductive alkylation. One μg of protein in 10 μl containing the cytotoxic activity was dissolved in 100 mM sodium hydrogen carbonate, and 1 μl of 45 mM dithiothreitol (DTT) was added into the protein solution. The mixture was incubated at 55°C for 15 min and the reaction was terminated by the addition of 1 μl of 100 mM iodoacetamide. After incubation of the products at room temperature for 15 min, 1 μl of trypsin (0.1 mg/ml in 100 mM sodium hydrogen carbonate solution) was added, and the mixture was incubated at 37°C for 3 hr. The reaction was terminated by the addition of 1 μl of 0.1% trifluoroacetic acid. The reaction products were kept at -80°C until the mass spectrometry analysis.

The LC system used was an Agilent Technologies Series 1200 system (Agilent, USA) equipped with a nanoflow pump (G2226A) as an analytical pump, a capillary pump (G1376A), a degasser (G1379B), an autosampler (G1377A), a chip cube interface (G4240A) and a ALS Thermo (G1330B). Chromatographic separations were performed on a ZORBAX 300SB-C18 column (0.075 μm × 43 mm, 5 μm), and the column temperature was maintained at 25°C. A gradient from mobile phase A (0.1% formic acid) to mobile phase B (0.1% formic acid/acetonitrile) was adopted. The sample injection volume and the flow rate were 40 nl and 300 nl/min, respectively. The LC program was as follows: 0.0 min, 5% B; 0.0–60.0 min, 5% to 60% B; 30.0–30.1 min, 60% to 90% B and; 30.1–40 min, 90% B. The stop time was 55 min. Detection was carried out with an Agilent 6530 instrument equipped with an Electrospray™ ionization source, controlled by the OpenLAB software program (Agilent, USA). Positive ionization was performed, and the following parameters were used: drying gas, N2 (5 L/min); drying gas temperature, 325°C; MS range, 350–2000 (MS) and 50–3000 (MS/MS); fragmentor (V), 175V; capillary voltage, 1800V; and acquisition mode, AutoMSMS. The mass spectroscopy analysis was carried out at Agilent Technologies Inc. (Tokyo, Japan). Spectrum Mill, a computer software program, was used for the determination and identification of peptide sequences from the mass spectroscopy data.

### Expression of mRNA for the new enterotoxin analyzed by RT-PCR

Total RNA was extracted from the *C*. *perfringens* isolates originated from the four outbreaks. The isolates were cultured in BHI medium and the cell bodies were harvested by centrifugation at 5,000 x g for 10 min, and the total RNA of the cells was extracted using a RiboPure-Bacteria Kit (Ambion, Thermo Fisher Scientific) according to the manufacturer's protocol. The isolated total RNA (2 μg) was reverse-transcribed using random primers with the High-Capacity cDNA Reverse Transcription Kits (ABI) according to the manufacturer's protocol, and was subsequently amplified by PCR using the Ex Taq HS (Takara).

The RT-PCR reaction mixture included 2 μl of the reverse transcriptase-reaction solution, 1×Ex Taq buffer, 200 μM dNTPs, 1 μM sense primer, 1 μM anti-sense primer and 1.25 units Ex Taq HS in a total volume of 50 μl. RT-PCR was performed using a GeneAmp PCR System 9700 (Applied Biosystems). The PCR conditions were: 10 min at 95°C, then 30 cycles of denaturation at 95°C for 30 sec, annealing at 60°C for 30 sec, and extension at 72°C for 1 min, followed by 10 min at 72°C. The specific sense and anti-sense primers for *cpile-a* were 5’-CAATGGGGCGAAGAAAATTA-3’ and 5’-GTTTCCTCTTAGCAAAAGCTGA-3’, respectively. The specific sense and anti-sense primer for *cpile-b* were 5’-TTGCAGTTCAGTCTGAGAAACC-3’ and 5’-CAGGGGAATTCGTATAATCTGC-3’, respectively.

### cDNA cloning, expression and purification of CPILE

A cDNA encoding the two components was amplified by PCR using KOD plus DNA polymerase (TOYOBO). The forward and reverse primers for *cpile-a* were 5’-GGATCCATGTTAGACGATAACCGACCTATG-3’ and 5’-GTCGACCTATATTAAAGTAGCATCAATAAT-3’, respectively. The forward and reverse primers for *cpile-b* were 5’-GGATCCATGATAAATAATACTTTTTTTATG-3’ and 5’-GTCGACCTAAAAAGGGTATTCAAGCACAAT-3’, respectively. Both forward primers contained a *BamH*I restriction site (underlined) at the 5’ end, and both reverse primers contained a *Sal*I restriction site (underlined). The PCR products were purified using the illustra GFX PCR DNA and Gel Band purification kit (GE Healthcare). The purified DNA fragments were sub-cloned into pCR-TOPO-XL (Invitrogen).

These cDNAs containing sub-cloned vectors digested using *BamH*I/*Sal*I were ligated into a pGEX 4T-2 vector that expressed a glutathione S-transferase (GST)-fusion protein, including a thrombin site at the N-terminus. The expression vectors were transformed into *E*. *coli* BL21 star competent cells (Invitrogen). Transformed *E*. *coli* were plated onto LB agar plates containing ampicillin (100 μg/ml). Single colonies of *E*. *coli* with homologue a/pGEX-4T-2 and homologue b/pGEX-4T-2 were picked up and inoculated into LB broth containing ampicillin (100 μg/ml) and were grown overnight at 37°C with shaking (150 rpm). A 4% volume of the overnight culture was then inoculated into LB broth containing ampicillin. The broth was cultured at 37°C until the absorbance of the culture at 600 nm reached to 0.6, then isopropyl β-D-thiogalactoside (IPTG, final concentration of 1 mM) was added. The cultivation continued for another 24 hr at 16°C with shaking. The *E*. *coli* cells transformed with cpile-a/pGEX4T-2 were harvested by centrifugation at 10,000 rpm for 10 min at 4°C. Proteins were extracted from the *E*. *coli* cells with the Bugbuster® protein extraction reagent (Novagen). After centrifugation at 9,000 x g for 30 min at 4°C, the supernatant was mixed with GSH Sepharose 4B beads (GE healthcare) overnight at 4°C with gentle agitation. The beads were washed in PBS by centrifugation at 1,000 x g for 10 min at 20°C, and then were suspended in a small volume of PBS. Thrombin (GE Healthcare) was added into the suspension of the beads and the suspension was agitated at room temperature for 30 min. The supernatant resulting from centrifugation of the sample at 1,500x g for 10 min was harvested and a suspension of benzamidine beads (Sigma-Aldrich) was added into the supernatant to remove thrombin.

The *E*. *coli* transformed with cpile-b/pGEX-4T2 were harvested and proteins were extracted from the *E*. *coli* with the Bugbuster® protein extraction reagent. After centrifugation at 9,000 x g for 30 min at 4°C, the supernatant was mixed with GSH-Sepharose 4B beads (GE healthcare) overnight at 4°C with gentle agitation. The beads were packed in a column and then exhaustively washed in PBS. The rCPILE-b was eluted from the beads with 10 mM glutathione, and treated with trypsin at 37°C for an appropriate period. The ratio of rCPILE-b:trypsin was 10:1 (w:w). The rCPILE-a and the trypsin-treated rCPILE-b were further purified by gel filtration using a Superdex 200 column. The final preparation of rCPILE-a and -b was checked for their purity by SDS-polyacrylamide gel electrophoresis (SDS-PAGE).

#### Measurement of NADase activity for the recombinant CPILE-a

The rCPILE-a (50 μl) was incubated for 6 hr at 37°C in the presence of 1 mM NAD^+^, 10 mM EDTA, 10 mM DTT, and 100 μg of bovine serum albumin in 40 mM Tris-HCl buffer (pH 7.5). The reaction was terminated by the addition of 40 μl of 0.1% formic acid in acetonitrile. Ten μl of an internal control solution (0.5 mM diethylnicotinamide/50% acetonitrile) was added into the reaction products. The final products were cleared by filtration using a10-kDa cut-off Nanosep® and Nanosep MF Centrifugal Devices (Pall, Port Washington, USA).

Nicotinamide (NA) and diethylnicotinamide were quantified by a 3200 Q TRAP LC/MS/MS System (AB Sciex, Foster City, CA, USA), equipped with an ESI source and a LC-20A Series HPLC System (Shimadzu Corporation, Kyoto, Japan). The HPLC conditions were: column, Poroshell 120 EC-C18, 2.1 mm i.d. × 50 mm, 2.7 mm (Agilent Technologies, Palo Alto, CA, USA); mobile phase, 10 mM ammonium acetate/acetonitrile, hold at 5% acetonitrile for 1 min, linear gradient of 5–90% acetonitrile in 6 min, hold at 90% acetonitrile for 1 min; retention time, NA 0.6 min and diethylnicotinamide 3.2 min. The MS parameters were as follows: source polarity, positive; source temperature, 300°C; ionization voltage, 5500 V; curtain gas, 20 psi and nebulizer gas, 70 psi. The ion transitions: parent > daughters (quantifier, Q; identifier, I) were as follows: nicotinamide, 123 [M+H]^+^ > 80 (Q), 78 (I) and diethylnicotinamide, 179 [M+H]^+^ > 108 (Q), 72 (I).

#### Assay of the ADP-ribosyltransferase activity for the recombinant CPILE-a

Vero and L929 cells were seeded at 5 × 10^5^ cells/dish into 60-mm culture dishes, and then were incubated for 24 hr in a CO_2_ incubator. After incubation, the cells from two plates were washed with PBS, scraped with a rubber policeman in PBS, and then harvested by centrifugation at 1,200 rpm for 10 min. The cells were suspended in 200 μl of 10 mM phosphate buffer (pH 8.5), and then sonicated to obtain homogenate proteins. Ten μg of the homogenate protein was incubated with 500 ng of the rCPILE-a for 60 min at 37°C in 50 μl of a reaction mixture containing 10 mM thymidine, 10 mM nicotinamide, 10 mM DTT, 5 mM MgCl_2_ and 10 μM ^[32P]^NAD (92.5 kBq, GE Healthcare) in 100 mM Tris-HCl buffer (pH 8.5). Trichloroacetic acid (5.5 μl of 100% w/v) was added to the reaction mixture. The precipitates obtained by centrifugation (10,000 x g, 10 min, 4°C) were washed with ice-cold ethylether, solubilized in 67.5 mM Tris-HCl, pH 6.8, containing 1% SDS, 25 mM DTT and 20% glycerol, and subjected to SDS-PAGE. Radioactive bands were visualized by autoradiography with Fuji RX film (Fuji Film Co., Tokyo, Japan).

#### SDS-PAGE of the recombinant CPILE-b

The oligomerization activity of rCPILE-b was examined by SDS-PAGE under various conditions. The trypsin-treated rCPILE-b was denatured by heating at 95°C for 5 min in the presence or absence of 50 mM DTT as a reductant. The denatured products were subjected to SDS-PAGE. Denaturation of the rCPILE-b by incubation at 37°C for 30 min was also performed instead of 95°C-heating.

#### Cell culture and cytotoxicity assay

The Vero and L929 cells were purchased from the Human Science Research Resources Bank (Osaka, Japan). All cells were cultured in Dulbecco's modified Eagle's minimum essential medium (DMEM, Sigma-Aldrich) supplemented with 5% fetal bovine serum (FBS, Gibco), non-essential amino acids (Sigma-Aldrich), 50 unit /ml penicillin, and 50 μg/ ml streptomycin.

The Cell Counting Kit-8 assay (Doujin-kagaku, Kumamoto, Japan) was used to evaluate the cytotoxicity. In 96-well plates, cells were seeded in 100 μl D-MEM medium supplemented with 1% FBS, non-essential amino acid, 50 unit/ml penicillin, and 50 μg/ml streptomycin at 1×10^4^ cells/well. A serially diluted rCPILE-a or -b was added into the wells 16 hr atfter incubation. Ten μl of 2-(2-methoxy-4-nitrophenyl)-3-(4-nitrophenyl)-5-(2, 4-disulfophenyl)-2H-tetrazolium, monosodium salt (WST-8, in kit) was added to the wells after a further 24 hr incubation, and the culture was continued for 3 hr. The absorbance of the reaction products at 450 nm was measured by an iMark Microplate Reader (Bio-Rad). A titer of the toxic effects was designated as U, based on the dilution factor showing the cytotoxicity.

#### Observation of the morphology of L929 cells

In 24-well plates, the cells (2 x 10^4^ cells/well) were seeded in 100 μl DMEM medium supplemented with 1% FBS, non-essential amino acids, 50 unit/ml penicillin, and 50 μg/ml streptomycin. The recombinant CPILE was added into the wells at 10^−7^, 10^−8^, or 10^−9^ M 16 hr after incubation, and the culture continued for another 24 h. The morphological changes of L929 cells were observed by optical microscopy.

#### Rabbit ileum loop test

Rabbits (Japanese white, male, 2.0 kg) were fasted for 24 hr before the operation. Various combinations of rCPILE-b and the trypsin-treated rCPILE-b (0.1 μg:0.9 μg, 1 μg:9 μg, and 10 μg:90 μg) were prepared in 1.0 ml of PBS and injected into a loop of the ileum. The doses of rCPILE were based on a previous study [25]. Cholera toxin (Wako Chemicals, Osaka, Japan) was injected as a positive control (1 μg/loop). The fluid accumulation ratio (F/A ratio) was calculated based on the volume of fluid of the loop (ml)/length of the loop (cm). Experiments were humanely conducted under the regulation and permission of the Animal Experiment Committee of the National Institute of Health Sciences (NIHS), Tokyo, Japan. The committee required the verbal informed consent of the participants of this study. The name of all participants in this study was included in the application documents to do experiments. The committee reviewed a design of study and contents of the experiment, approved them. The committee interviewed one of the authors (YK), then permitted us to do this study. The committee documented all of information according to this study. Five rabbits were used to demonstrate the enterotoxic activity of the test materials, including the preliminary test. All rabbits were carefully taken care of according to the guidelines of the NIHS. The rabbits were anesthetized with sodium pentobarbital (Somnopentyl, Kyoritsu Seiyaku Corporation, Tokyo) and opened the abdomen, injected the test material into a loop (approximately 5 cm) of the ileum. After closing the abdomen, the rabbits were stayed in a clean cage under careful observation. The rabbits were awoken from anesthesia within 1 hr. The rabbits were euthanized by the injection of a large volume of sodium pentobarbital 18 hr after the injection of the test material.

#### Antibodies against recombinant CPILE-a and -b and the Western blot analysis

Anti-serum against CPILE-a and the trypsin-treated rCPILE-b was prepared in rabbits at Funakoshi Co., Ltd (Tokyo, Japan). The materials, which were precipitated by 60%-ammonium sulfate saturation, from the cultures incubated strain W5052 in modified DS medium were used as a test material. A western blot analysis was performed after protein separation on SDS-PAGE gels, followed by electrophoretic transfer to Immun-Blot PVDF membranes (Bio-rad) using a Trans-Blot SD Semi-Dry Electrophoretic Transfer Cell system (Bio-rad). The detection of the homologues was accomplished by incubating the membrane with a blocking buffer (10% Skim milk in PBS, BD) overnight at 4°C. The membrane was then incubated with 1,000-fold diluted polyclonal antiserum in Tris-buffered saline (TBS) containing 0.1% Tween-20 (TBST) for 1 hr. After being washed, the membrane was treated with goat anti-rabbit IgG coupled with horseradish peroxidase (Pierce) in TBST for 1 hour. The reaction was visualized by the use of a Metal Enhanced DAB Substrate Kit (Pierce) according to the manufacturer's instructions.

## Results

### Genomic sequencing and bioinformatics of *C*. *perfringens* strain W5052

We performed mass sequencing on the total DNA isolated from strain W5052, using the Roche/454 next generation sequencer. From 220,012 total raw reads, 132 contigs were assembled. The total aggregate length was 3,336,496 nt. Since the length of 112 of total contigs was 500 nt or longer, the sequencing was valuable to analyze the genomic DNA. A comparison between the contigs of strain W5052 and the complete genome DNA sequence of CPE-producing *C*. *perfringens* strain SM101 (DDBJ/EMBL/FASTA accession number: BA000016) was performed, and 21 W5052 strain-specific contigs were obtained. The Jemboss software program indicated that 54 genes were predicted to be present in the 21 contigs. After translation of the 54 genes to amino acid sequences, 45 amino-acid sequences were determined to be bacteriophage-related homologues of *C*. *botulinum*, as judged by a Blast search. Since the 9 residual amino-acid sequences remained as unknown genes, these 9 genes were considered to be the candidates for the new enterotoxin gene(s). Iota toxin homologue components a (ia) and b (ib) of *C*. *perfringens* were found in contig No. 31.

### Partial purification of a new enterotoxin from the culture of *C*. *perfringens* strain W5052

**Fig 1:**
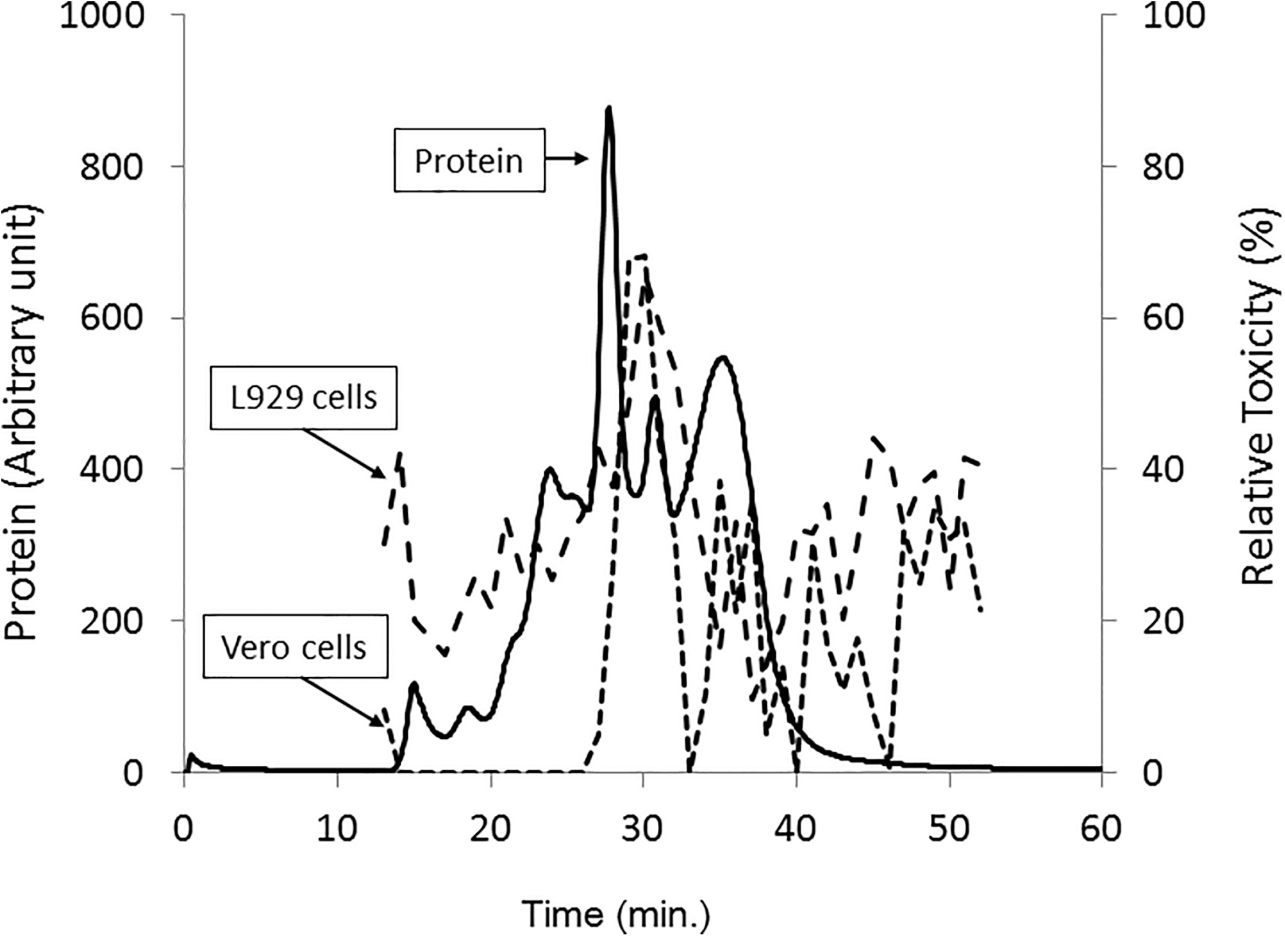
Gel filtration of a new enterotoxin produced by *Clostridium perfringens* strain W5052. The elution profile of the proteins containing a new enterotoxin is indicated with a solid line. The relative toxicity of the fraction to L929 and Vero cells was indicated with dot and dashed lines, respectively. The relative toxicity (%) was determined with the Cell counting Kit-8 (DOJINDO, Kumamoto, Japan).

A new enterotoxin was partially purified from the cultured modified-DS medium by ammonium sulfate fractionation. The fraction of 0–70% saturation showed cytotoxicity to both Vero and L929 cells (Table 1). On the other hand, the 0–40% and 60–70% fractions were cytotoxic only to Vero cells, while the factions of 40–50% and 50–60% were also cytotoxic to both cell lines. These findings suggested that both the 40–50% and 50–60% ammonium sulfate fractions contained the new enterotoxin(s). A mixture of fractions 40–50% and 50–60% was subjected to gel filtration using a Superdex 200 column. Six major protein peaks were found (Fig 1). The cytotoxicity to both cell lines was concentrated in fraction No. 30, suggesting the presence of the new enterotoxin(s) in that fraction.

**Table 1:** Cytotoxic activity in the ammonium sulfate precipitation fractions of a new enterotoxin produced by *Clostridium perfringens* strain W5052.

Fraction of ammonium sulfate precipitation	Protein concentration (mg/ml)	Toxicity* (U/ml)	Specific toxicity (U/mg)	Toxicity* (U/ml)	Specific toxicity (U/mg)
0–70%	23	6	0.26	1	0.04
0–40%	27	11	0.41	N.D^.^ **	N.D^.^ **
40–50%	11	14	0.13	9	0.08
50–60%	110	14	0.13	11	0.10
60–70%	77	10	0.13	N.D.**	N.D^.^ **

*:Toxicity unit (U) was designated, based on the dilution factor showing the cytotoxicity determined, using Cell-counting Kit.

**:Not detected.

### Analysis of the partially purified new enterotoxin by mass spectrometry

The Spectrum Mill software program was used to analyze the MS data for the partially purified new enterotoxin (fraction No. 30) to identify the proteins under the default settings. Sixty six proteins in total were found for the candidate of a new toxin. The six proteins with the highest credibility belonged to *C*. *perfringens*.

Predictive open reading frames were extracted from the sequence of the 132 contigs of W5052, and the frames were listed as a protein database. The database showed 9 predicted proteins. The proteins were: lipoprotein, flavoprotein, Fe-S cluster assembly protein, phosphotransferase, elongation factor, GroES, transcription elongation factor, 30S ribosomal protein S4 of *C*. *perfringens*, and components Sa and Sb of the iota-like toxin of *C*. *spiroforme*.

The MS data were analyzed again by the Spectrum Mill software under more sensitive conditions. Five distinct peptides of iota-like toxin component a and a peptide of iota-like toxin component b were found.

### Molecular biological properties of a candidate gene for a new enterotoxin of *C*. *perfringens* strain W5052

**Fig 2:**
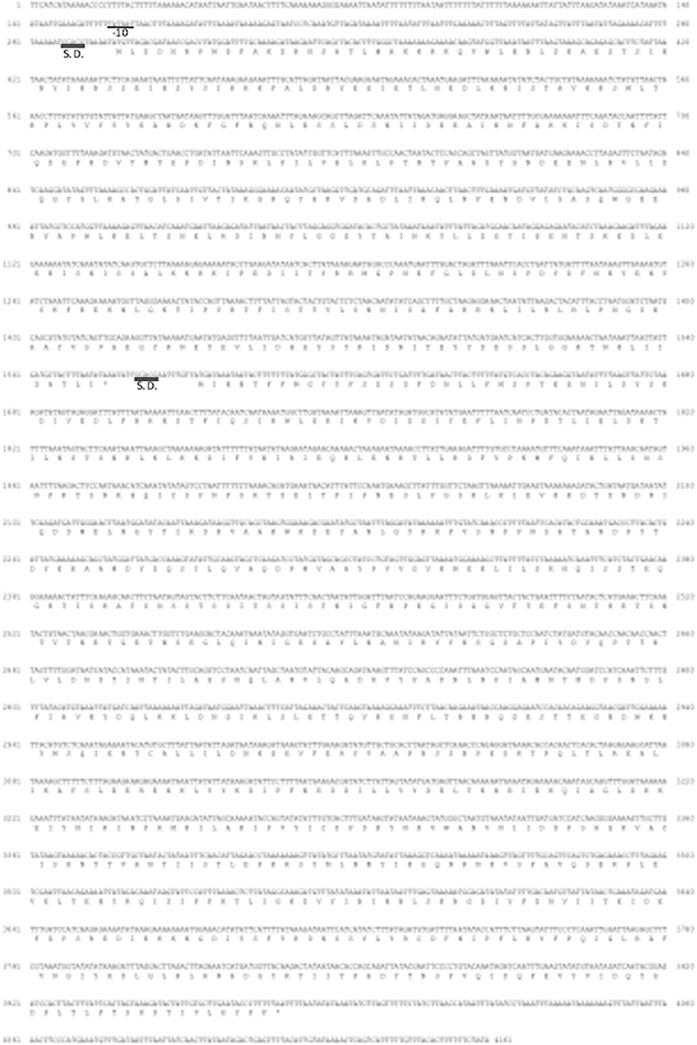
The nucleotide and amino acid sequences of the new enterotoxin components, CPILE-a and CPILE-b, of *Clostridium perfringens* strain W5052. A -10 region promoter sequence and the putative Shine Dalgarno (S. D.) sequence were presented by single and double underlining, respectively. The in-frame stop-codon is indicated by asterisks.

The 4161 nucleotide sequences contained two open reading frames in contig No. 3. One was CPILE-a, the other CPILE-b. The cDNA sequence of CPILE-a and -b, and their deduced amino acid sequences are shown in Fig 2 (DDBJ/EMBL/FASTA accession numbers AB921559 and AB921560). The gene of CPILE-a started at the initiation codon ATG at position 299 and ended at the stop codon at position 1556. A consensus ribosome binding like site, GGAGG, was located six nucleotides upstream of the initiation codon. DNA stretches of TATAAT, the -10 *Clostridium* consensus promoter regions [26], were identified between position 158 and 163 (underlined, Fig 2). The gene of CPILE-b, from the initiation codon ATG (position 1577) to the stop codon at position 3974, was preceded by a consensus ribosome binding-like site (GGAGG) between positions 2864 and 2868. The signal peptide sequence region of CPILE-a and CPILE-b was not conserved in the genome of strain W5052. No promoter consensus sequences were found on the 18 noncoding nucleotides between the two genes.

### Alignment of CPILE and other iota toxin group members

**Fig 3:**
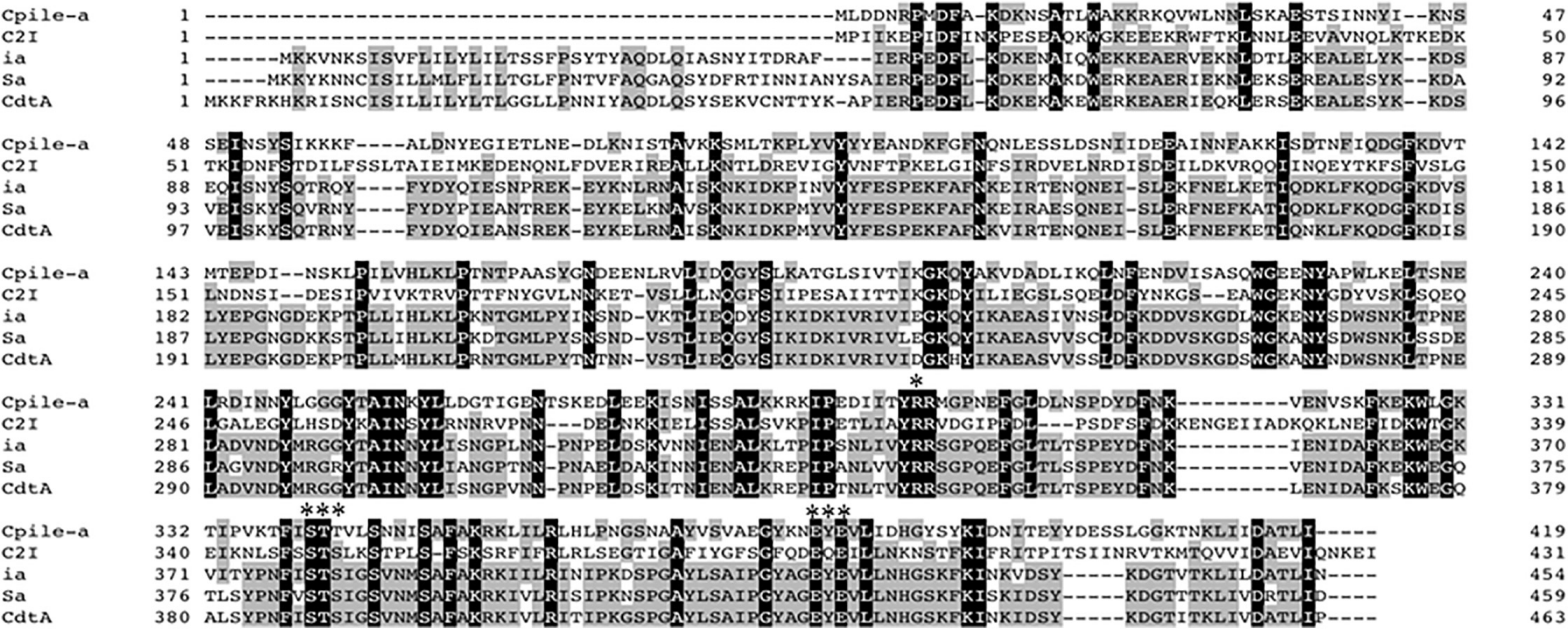
The alignment of the amino acid sequences of component a of the new enterotoxin, CPILE-a, of C*lostridium perfringens* strain W5052. The amino acid sequence of the component a homologue was compared with those of C2I (C2 toxin Component I of *C*. *botulinum*, DDBJ Accession Number: AJ224480), ia (iota toxin ia of *C*. *perfringens*, DDBJ Accession Number: X73562), Sa (iota like toxin Sa of *C*. *spiroforme*, DDBJ Accession Number: X97969), and cdtA (ADP-ribose transferase enzymatic component of *C*. *difficile*, DDBJ Accession Number: L76081). Dashes show the gap used to maximize the identity. Black shading indicates identical amino acid residues among the 5 proteins. Gray shading indicates more than 60% identical amino acid residues among the 5 proteins. In NADase, three conserved regions, aromatic residue-R, STS motif, and EXE motif, indicated by asterisks.

The nucleotides in the coding region of CPILE-a corresponded to 419 amino acid residues. The calculated molecular weight was 47,470.66. The deduced amino acid sequences of the component a homologue are shown in Fig 3. The amino acid sequence of CPILE-a was highly homologous to that of ADP-ribose transferases, such as iota-like toxin Sa of *C*. *spiroforme*, C2 toxin component I of *C*. *botulinum*, iota toxin ia of *C*. *perfringens*, and ADP-ribosyltranferase cdtA of *C*. *difficile*. CPILE-a showed an amino acid sequence identity to that of ia (44.0%), cdtA (43.3%), Sa (43.1%), and C2 I (28.9%), and similarity to that of Sa (83.0%), ia (82.5%), cdtA (82.7%) and C2 I (77.7%). The arginine residue as the catalytic center and a Glu-X-Glu (EXE) motif were completely conserved among CPILE-a of W5052, and other ADP-ribose transferase homologues. The sequence of the STS motif of CPILE-a was 100% similarity to the other ADP-ribose transferase homologues.

**Fig 4:**
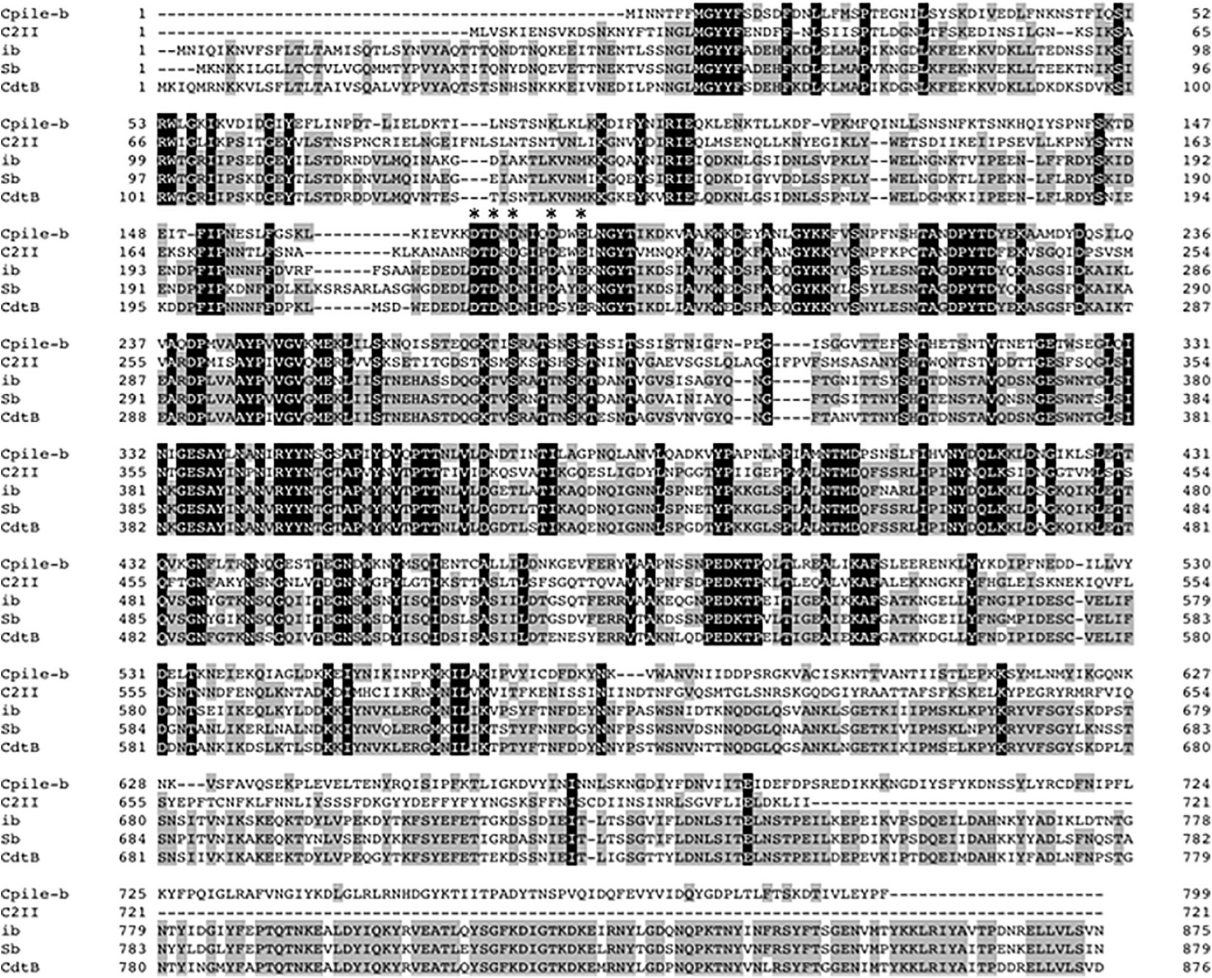
The alignment of amino acid sequence of component b of the new enterotoxin, CPILE-b, of *Clostridium perfringens* strain W5052. The amino acid sequence of the component b homologue was compared with those of C2II (C2 toxin component II of *C*. *botulinum*, DDBJ Accession Number: D88982), ib (iota toxin ib of *C*. *perfringens*, DDBJ Accession Number: X73562), Sb (iota-like toxin Sb of *C*. *spiroforme*, DDBJ Accession Number: X97969), and cdtB (ADP-ribosyltranferase binding component of *C*. *difficile*, DDBJ Accession Number: L76081). The dashes show the gap used to maximize the identity. Black shading indicates the identical amino acid residues among the 5 proteins. Gray shading indicates more than 60% identical amino acid residues among the 5 proteins. Asterisks represent the amino acid residues predicted to be involved in the interaction of the enzymatic component in the presence of Ca^2+^ (23).

The nucleotides of CPILE-b gene contained a coding region corresponding to 799 amino acid residues. The calculated molecular weight was 91,143.18. The deduced amino acid sequence of homologue b is shown in Fig 4. CPILE-b protein showed a sequence identical to C2 toxin component II (36.7%), ib (37.8%), Sb (38.3%), and cdtB (38.8%), and similar to that of C2 component II (81.0%), ib (77.6%), Sb (78.5%), and cdtB (77.7%). The cluster of aspartic acid residues, which form the site that interacts with the enzymatic component in the presence of Ca^2+^ [27], was completely conserved among all of the homologues (Fig 4).

### Expression of the mRNA of the homologue a and b genes

**Fig 5:**
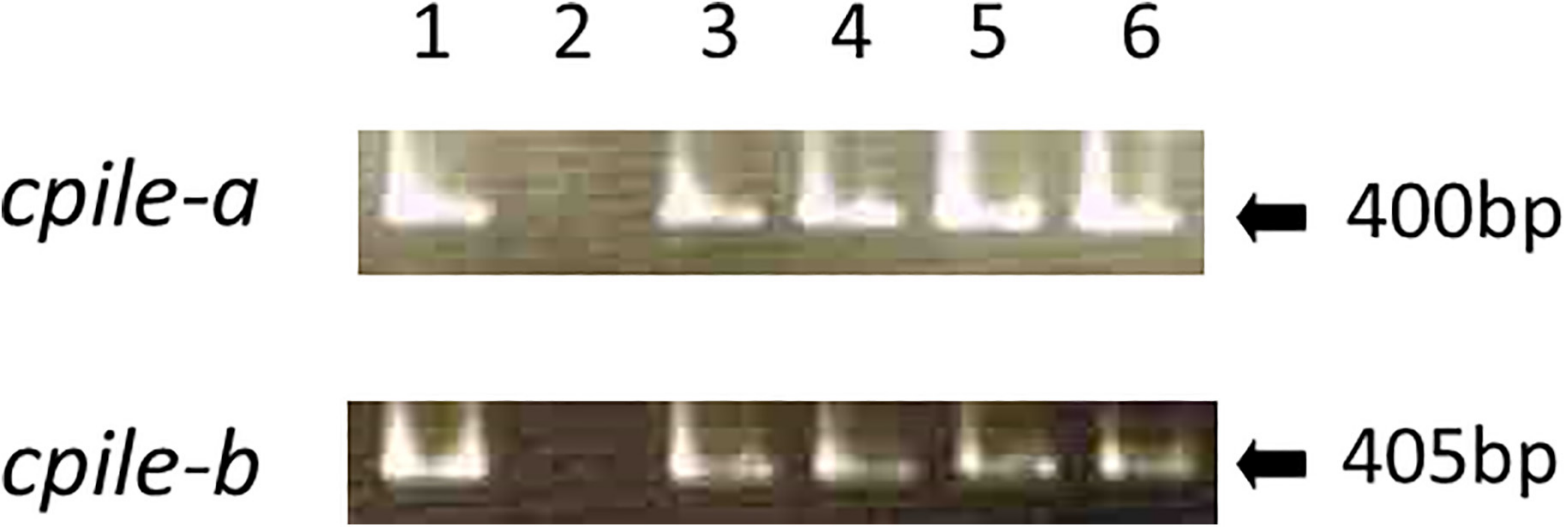
The expression of mRNA for the *cpile-a* and *-b* of *Clostridium perfringens strain* W5052. The genes of CPILE-a and -b in the isolates of the four outbreaks were amplified by RT-PCR. Amplicons of 400 bp (*cpile-a*) and 405 bp (*cpile-b*) are shown. Lane 1, Tokyo 1997; Lane 3, Tokyo 2003; Lane 4, Osaka 2009; and Lane 5, Tochigi 2010). Lane 2 is a negative control (CPE-producing strain).

The four strains of *C*. *perfringens* isolated from the individual outbreaks were examined for the expression of *cpile-a* and *-b* genes. The amplicons of *cpile-a* (400 bp) and *cpile-b* (405 bp) were detected in the test samples by RT-PCR, but were not in the strain harboring the *cpe* gene (negative control) (Fig 5). The gene of CPILE-a and -b was detected at almost equal amounts in the amplicons in the four isolates. These results indicated that the isolates of *C*. *perfringens* originated from the four outbreaks expressed mRNA for *cpile-a* and *-b*.

### cDNA cloning and preparation of a recombinant protein of CPILE

**Fig 6:**
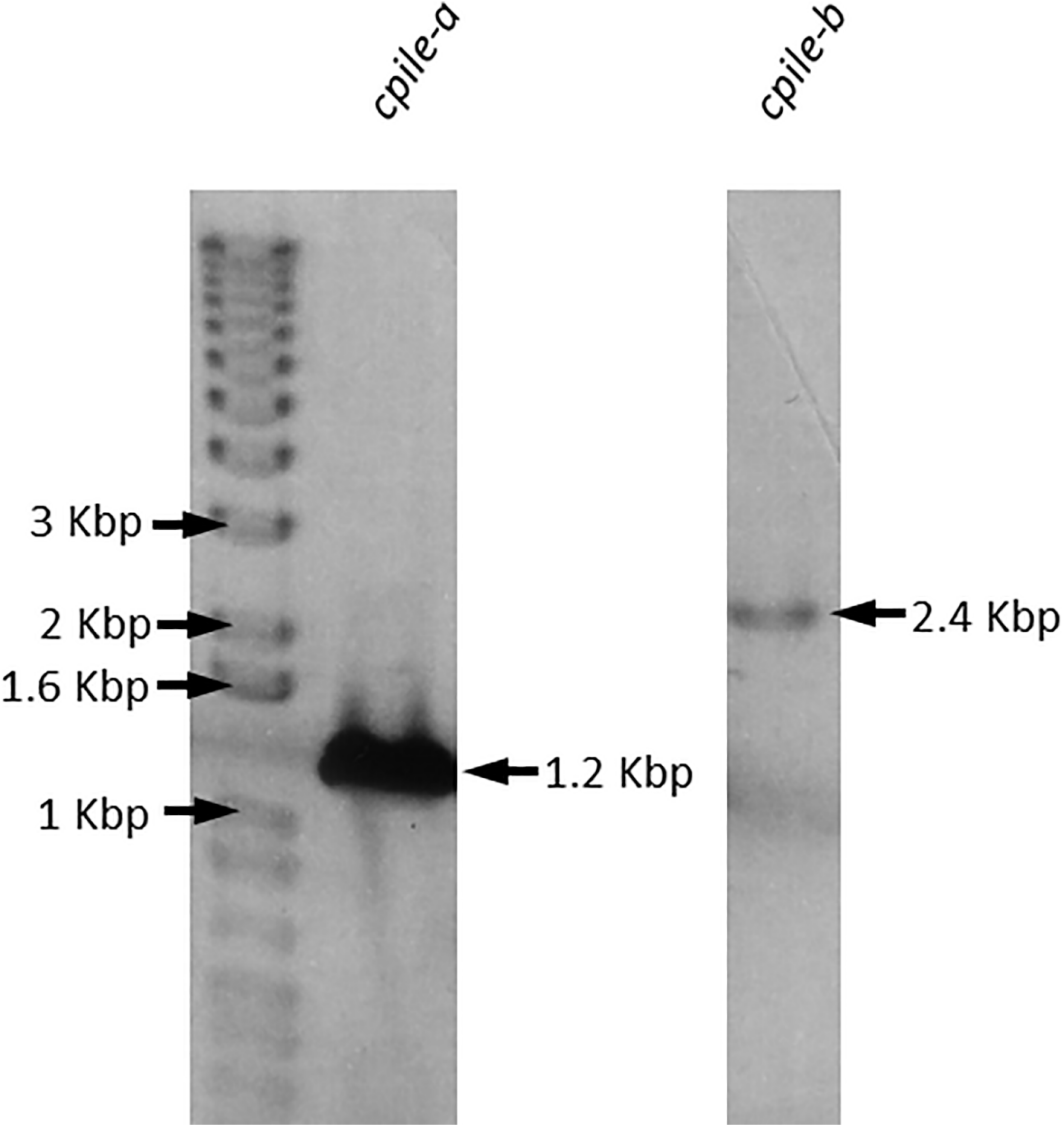
The cDNA cloning of *cpile-a and -b* of *Clostridium perfringens* strain W5052. The PCR products were analyzed by 1% agarose S gel electrophoresis. These amplified PCR fragments are indicated with arrows.

**Fig 7:**
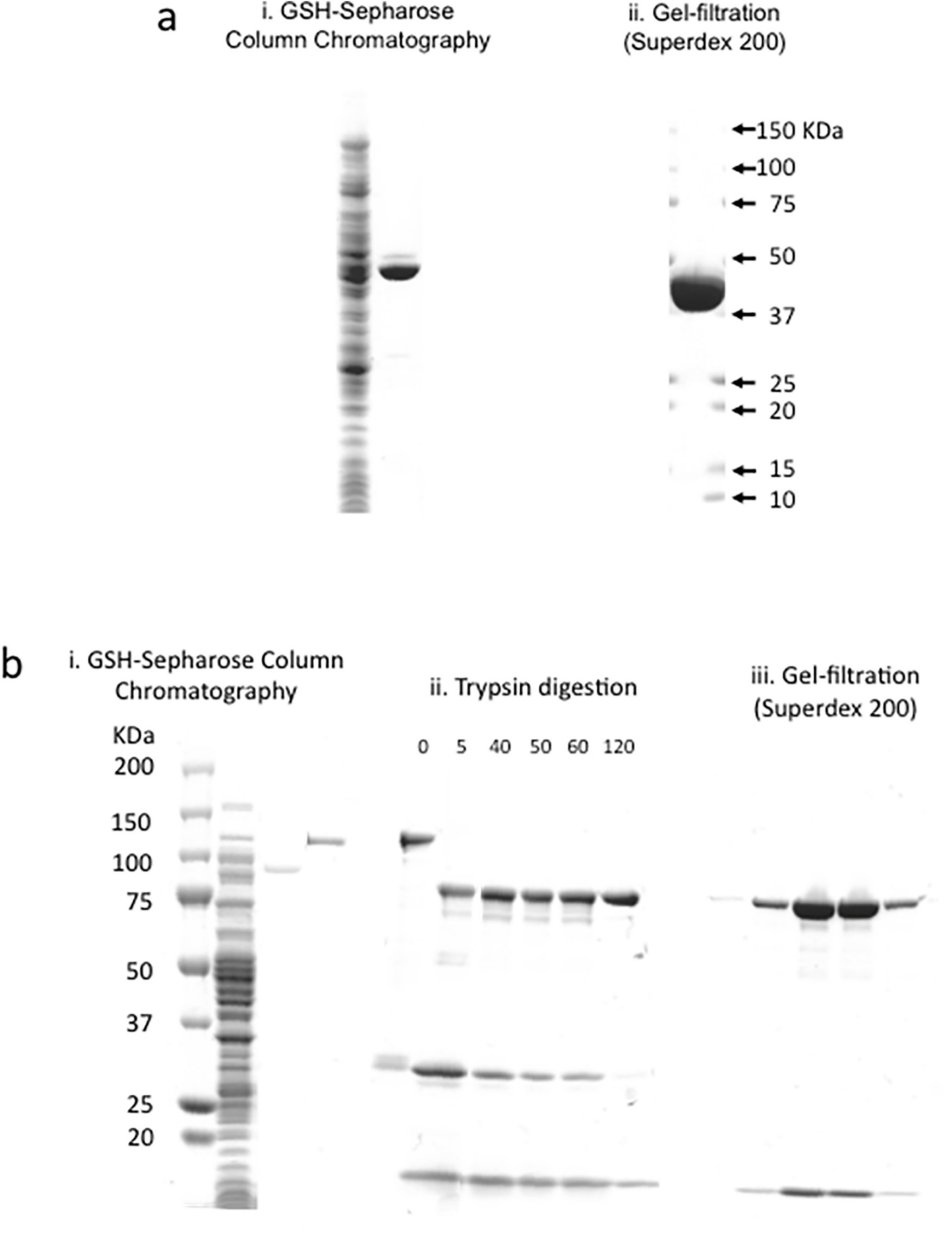
The purification of recombinant CPILE-a and -b of *Clostridium perfringens* strain W5052. a. Purification of the recombinant CPILE-a. Coomassie staining of the rCPILE-a after GSH-Sepharose column chromatography. The GST-fused rCPILE-b (1 μg) was treated with thrombin (i). A final product of the rCPILE-a (3 μg) after gel filtration using a Superdex 200 was analyzed (ii). b. Purification of the recombinant CPILE-b. Coomassie staining of the rCPILE-b after GSH-Sepharose column chromatography. The GST-fused rCPILE-b was treated with thrombin (i, lane 1). The GST-fused rCPILE-b (0.5 μg) was run in lane 3. The results of trypsin treatment of the GST-fused rCPILE-b were analyzed. Ten μl of the GST-fused rCPILE-b (100 μg) with trypsin (10 μg) mixture was electrophoresed (ii). The final product (10 μl of the fraction per lane) of the rCPILE-b using gel filtration of Superdex 200 column was analyzed (iii).

PCR was performed to obtain a full-length cDNA for *cpile-a* and *-b*, using the genomic DNA as the template. The cDNA of both components was generated as distinct single fragments of about 1200 and 2400 bp, respectively (Fig 6). The recombinant CPILE-a was successfully expressed as a GST-fusion protein in *E*. *coli* and was purified with apparent homogeneity by GSH-affinity chromatography and gel filtration (Fig 7A-i and 7A-ii). A yield of the rCPILE-a was 7 mg per 1-liter culture of *E*. *coli*. The rCPILE-b was also successfully expressed as a GST-fusion protein in *E*. *coli*. Due to resistance to thrombin cutting, the rCPIEL-b was purified as a GST-fused protein without contaminating materials (Fig 7B-i). The yield of the GST-fused rCPILE-b was 4 mg per 1-liter culture of *E*. *coli*. The GST-fused rCPILE-b was incubated in the presence of trypsin. The original band of the GST-fused rCPILE-b disappeared, and products with low (30 kDa) and high (75 kDa) molecular weights rapidly appeared (Fig 7B ii). The band at 75 kDa remained 120 min after incubation. The trypsin-treated rCPILE-b purified by gel filtration ran in the SDS-gel as a single band (Fig 7B iii).

### Characterization of CPILE produced by *C*. *perfringens* strain W5052

**Fig 8:**
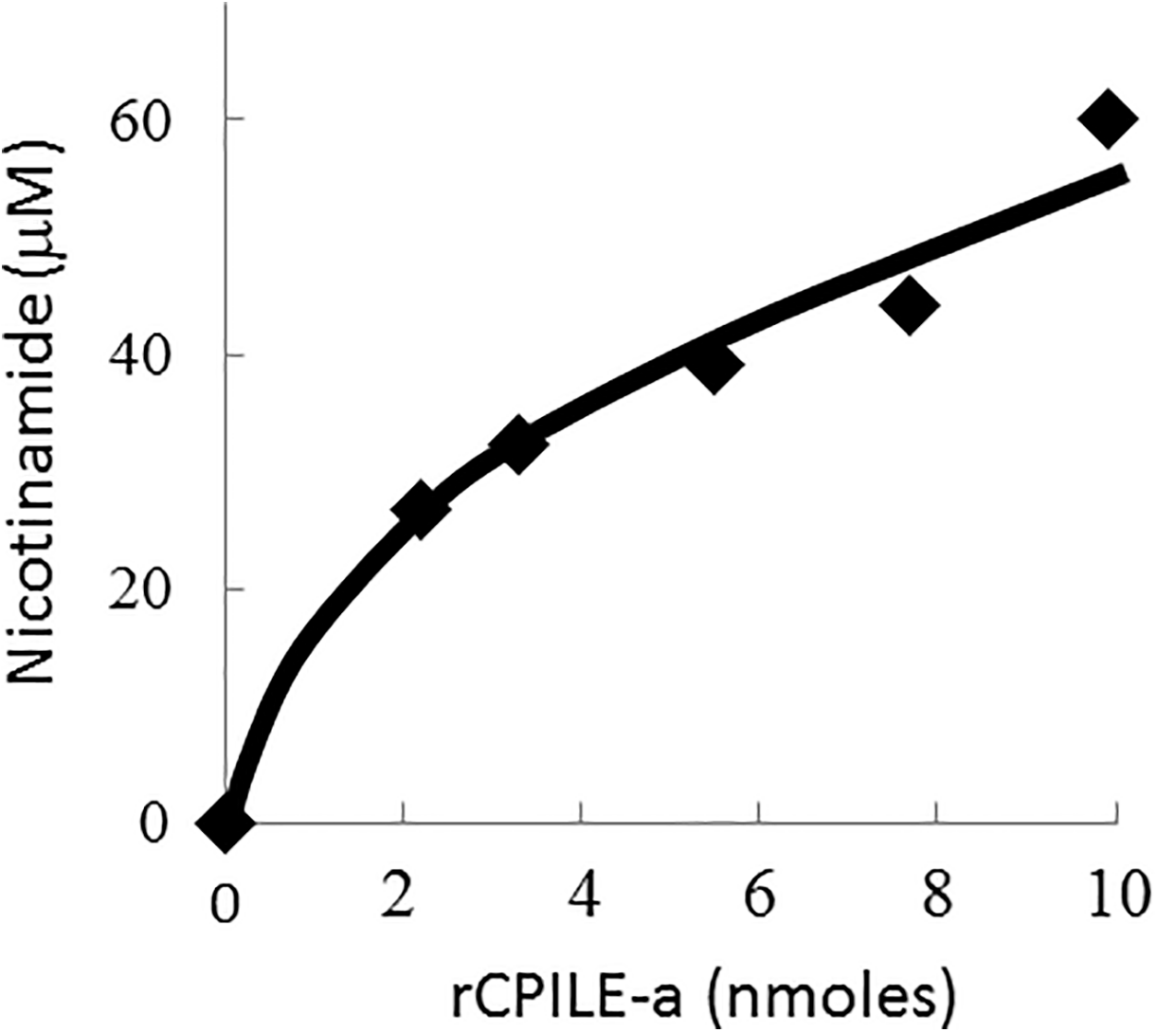
The NADase activity of the rCPILE-a of *Clostridium perfringens* strain W5052. Various concentrations of the recombinant component a homologue were incubated at room temperature for 6 hr with 1 mM NAD+, and the NA in the products was quantified by the MS system.

The ADP-ribosylation activity of the rCPILE-a was evaluated. The NA released from NAD was measured by a LC/MS/MS system to determine the NADase activity. The amount of the NA released in the CPILE-a test samples after the addition of NA was increased compared with that in the samples with only the rCPILE-a. The amount of the NA released was increased, depending on the dose of the rCPILE-a (Fig 8). The released NA was not detected in a mixture of NAD where the rCPILE-a was boiled for 10 min (data not shown). These findings suggested that the rCPILE-a possessed NADase activity.

**Fig 9:**
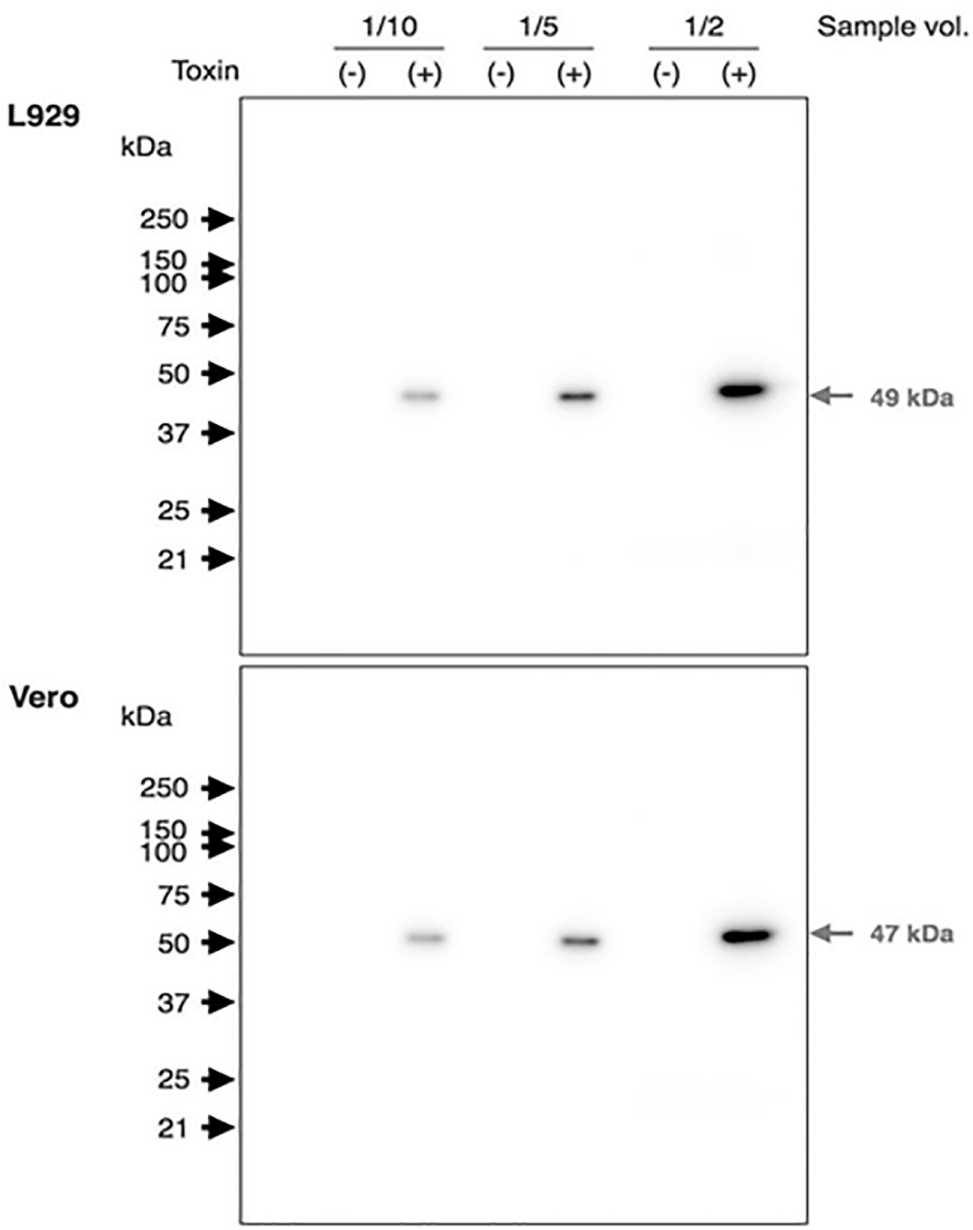
The ADP-ribosylation of the lysates prepared from Vero and L929 cells by rCPILE-a of *Clostridium perfringens* strain W5052. The cells were homogenized as described in the Material and Methods section. The cell lysates were treated with the rCPILE-a in the presence of ^[32P]^NAD and the reaction products were subjected to SDS-PAGE.

When the lysates from L929 and Vero cells were incubated with 500 ng of the rCPILE-a in the presence of 10 μM ^[32P]^NAD, a single radioactive band appeared in the SDS-PAGE with an apparent molecular mass of 47–49 kDa, corresponding to the molecular weight of actin monomer (Fig 9). Intensity of the band was dose-dependently increased by the application of the sample, indicating that the rCPILE-a possessed ADP-ribose transferase activity. These results demonstrated that the CPILE-a is an enzyme mediating ADP-ribosylation.

**Fig 10:**
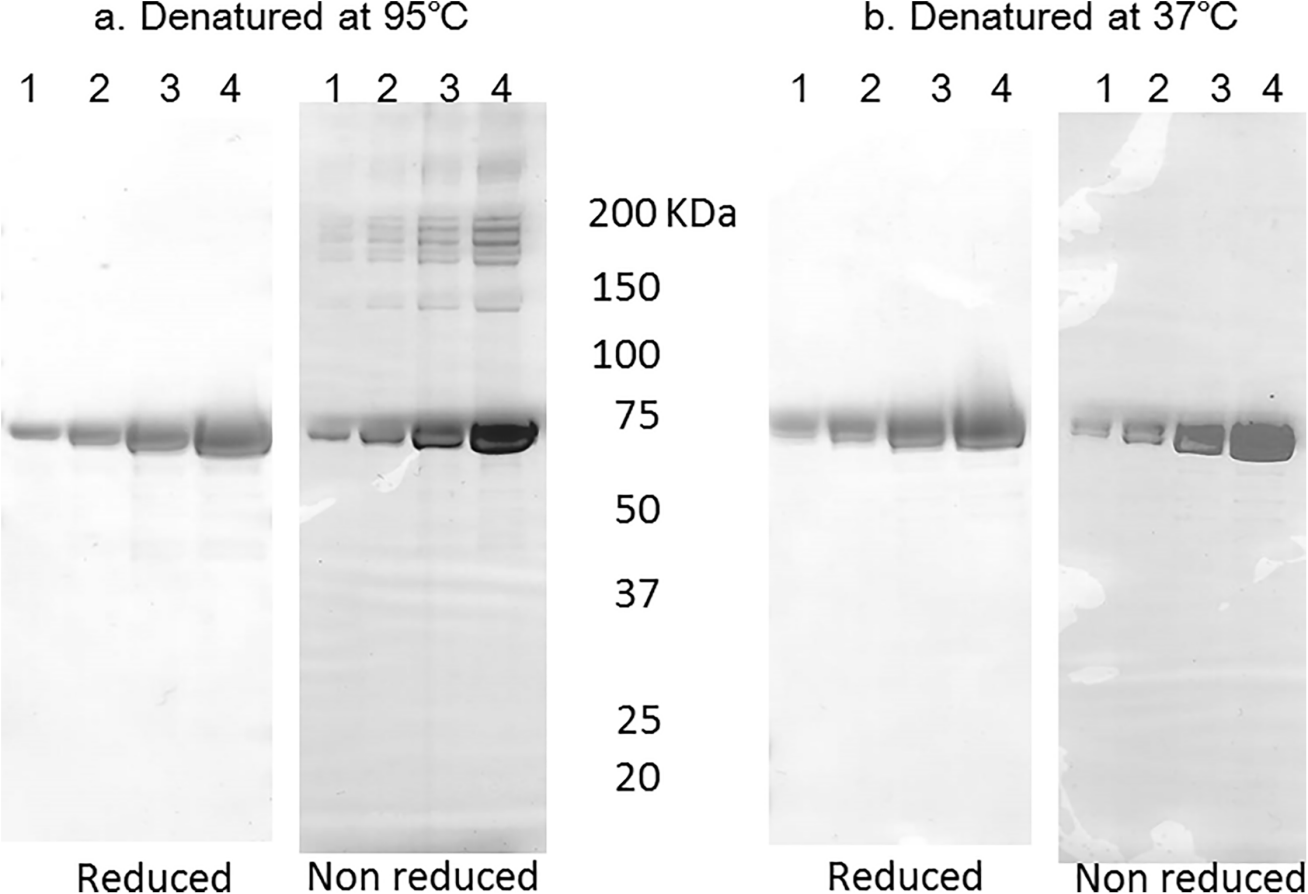
The electrophoretic properties of rCPILE-b of *Clostridium perfringens* strain W5052. The rCPILE-b was heated and subjected to SDS-PAGE with or without reduction. Lane 1, 0.5 μg of the recombinant b homologue; Lane 2, 1.0 μg; Lane 3, 2.0 μg; and Lane 4, 4.0 μg.

The electrophoretic properties of the rCPILE-b were then examined. The trypsin-treated rCPILE-b was heated at 95°C in the presence of SDS and then subjected to SDS-PAGE. Although the trypsin-treated rCPILE-b migrated into the gel as a monomer under the reduced conditions, many bands with a ladder form at the positions of higher molecular weight were detected under the non-reduced condition (Fig 10A). The heat denaturation of the trypsin-treated rCPILE-b without reduction induced oligomerization. Denaturation of the trypsin-treated rCPILE-b by incubation at 37°C did not induce oligomerization (Fig 10B). These findings are consistent with those of the iota toxin of *C*. *perfringens* and iota-like toxin of *C*. *spiroforme* [17, 28].

### The influence of CPILE on the viability and morphology of cultured cells

**Fig 11:**
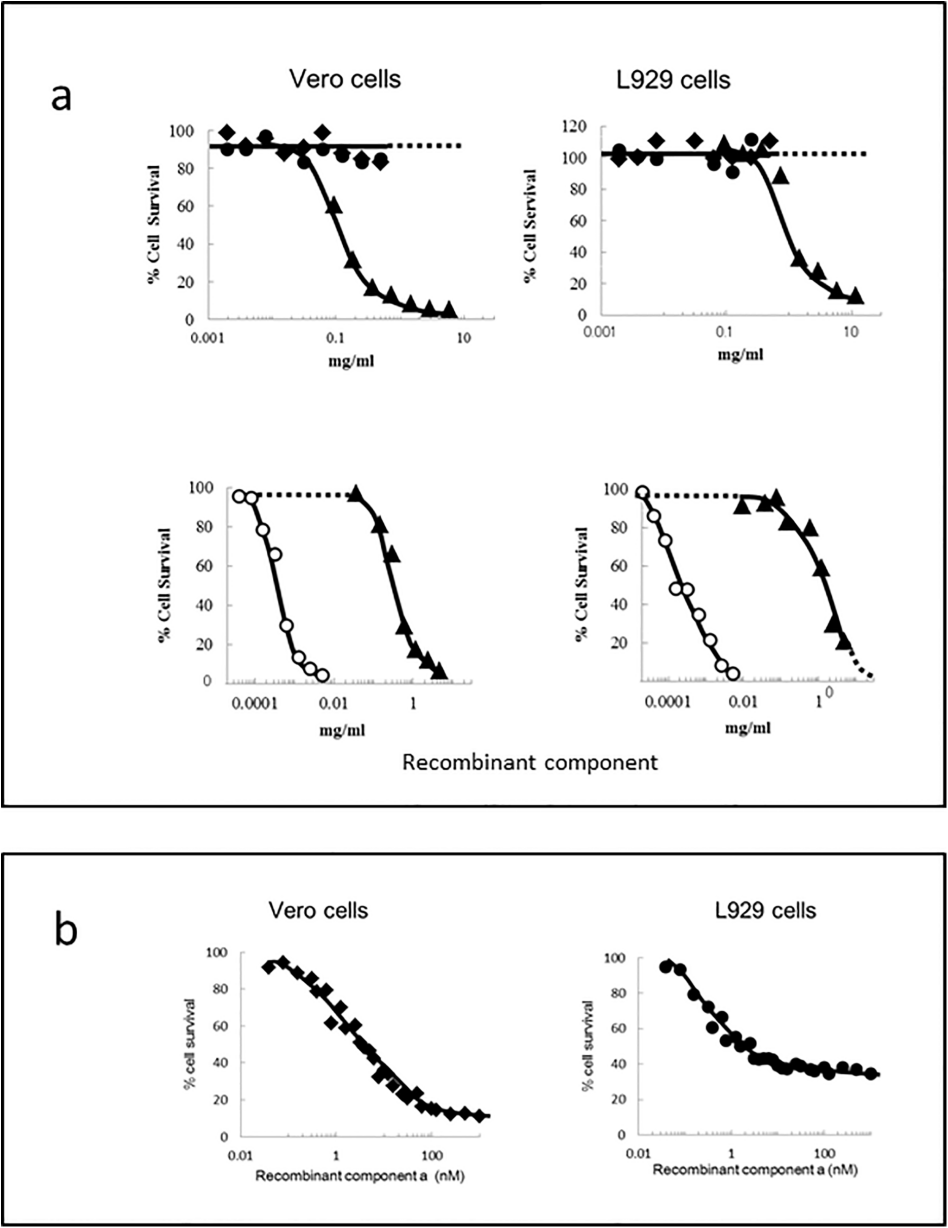
The cytotoxicity of the rCPILE of *Clostridium perfringens* strain W5052. a. Vero and L929 cells were treated for 16 h with a various concentrations of the rCPILE-a (◆), GST-fused rCPILE-b (●), trypsin-treated rCPILE-b (○), and the ammonium sulfate precipitation fraction (0–70%) (▲). The cell survival (%) was measured using a Cell Counting Kit-8. b. Vero cells (◆) and L929 cells (●) were treated with various concentrations of the rCPILE-a in the presence of 10 nM trypsin-treated rCPILE-b.

The rCPILE-a and GST-fused rCPILE-b did not show cytotoxic activity to either Vero or L929 cells, although the crude fraction (0–70% ammonium-sulfated precipitated fraction) killed both kinds of the cells (Fig 11A, top). The trypsin-treated rCPILE-b killed both kinds of cells at high concentrations, like the crude faction (Fig 11A, bottom). When the cells were incubated in the presence of 10^−9^ M of the trypsin-treated rCPILE-b neither the Vero nor L929 cells were left intact. When the rCPILE-a was added to the medium in the presence of 10^−9^ M of the trypsin-treated rCPILE-b, the viability of both kinds of cells decreased, depending on the amount of rCPILE-a added (Fig 11B). The rCPILE-a and trypsin-treated rCPILE-b were co-operatively functioned, thus suggesting that the CPILE of strain W5052 forms a binary toxin.

**Fig 12:**
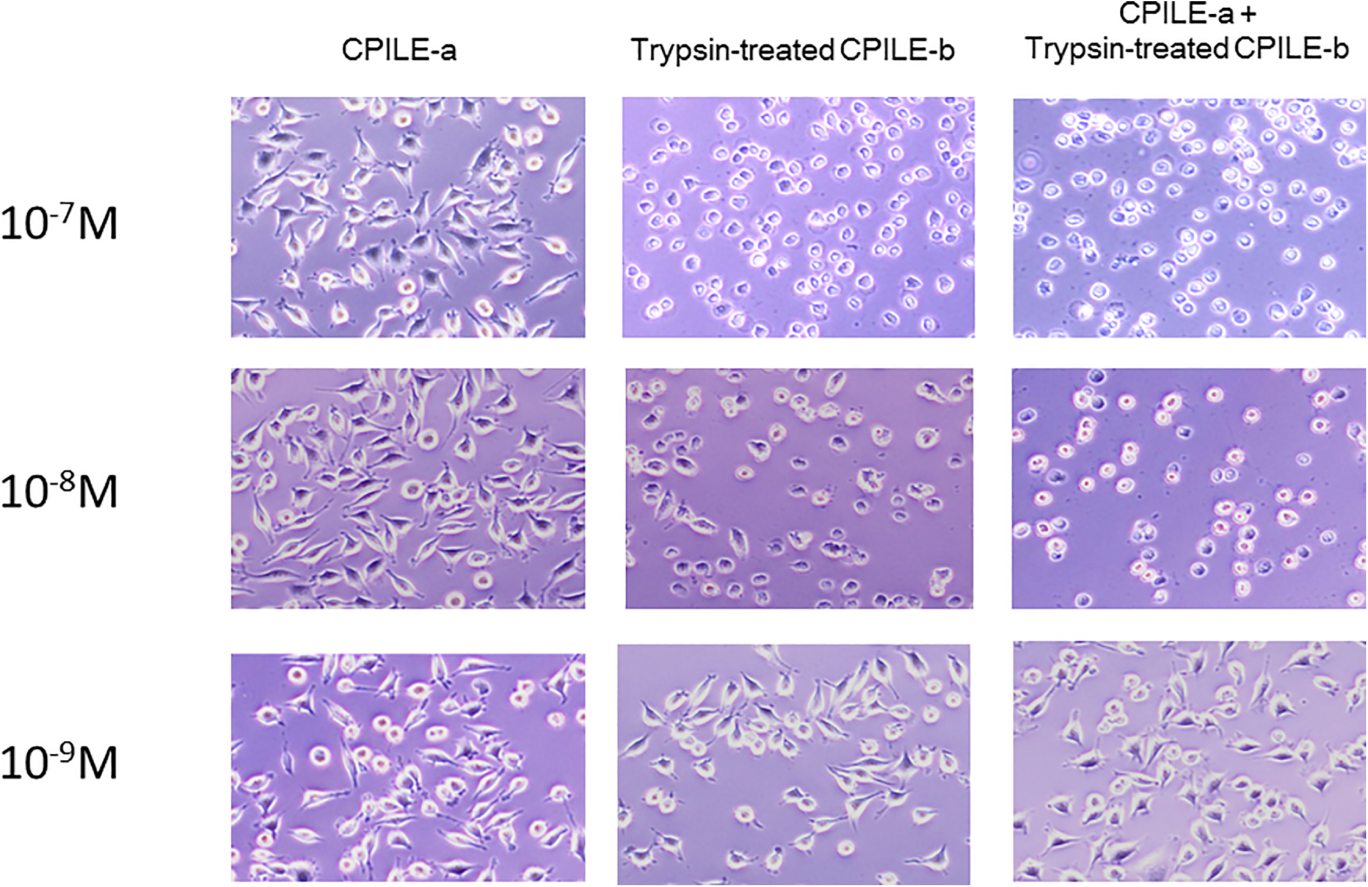
The morphological changes of the L929 cells after treatment with the rCPILE of *Clostridium perfringens* strain W5052. The L929 cell cells were treated for 16 hours with various concentrations of the rCPILE-a, trypsin-treated rCPILE-b, and a mixture of rCPILE-a and trypsin-treated rCPILE-b. The morphology of the L929 cells was observed by optical microscopy.

Treatment with a mixture of 10^−7^ M of rCPILE-a and trypsin-treated rCPILE-b killed the L929 cells more effectively in comparison to treatment with trypsin-treated rCPILE-b only, further supporting the binary effects (Fig 12). Treatment with 10^−8^ M of the mixture of rCPILE-a and trypsin-treated rCPILE-b affected the morphology of L929 cells, changing them from flat to rounded. The cells treated simultaneously with the rCPILE-a and trypsin-treated rCPILE-b showed a balloon-like cell shape with expanding cytosol, while the cells treated with the trypsin-treated rCPILE-b alone did not show this balloon-like shape. The balloon-like cell shape would have resulted from the disrupted permeability of the cells with dysfunction of the intracellular actins that were ribosylated by CPILE-a.

### Enterotoxic activity of CPILE

**Fig 13:**
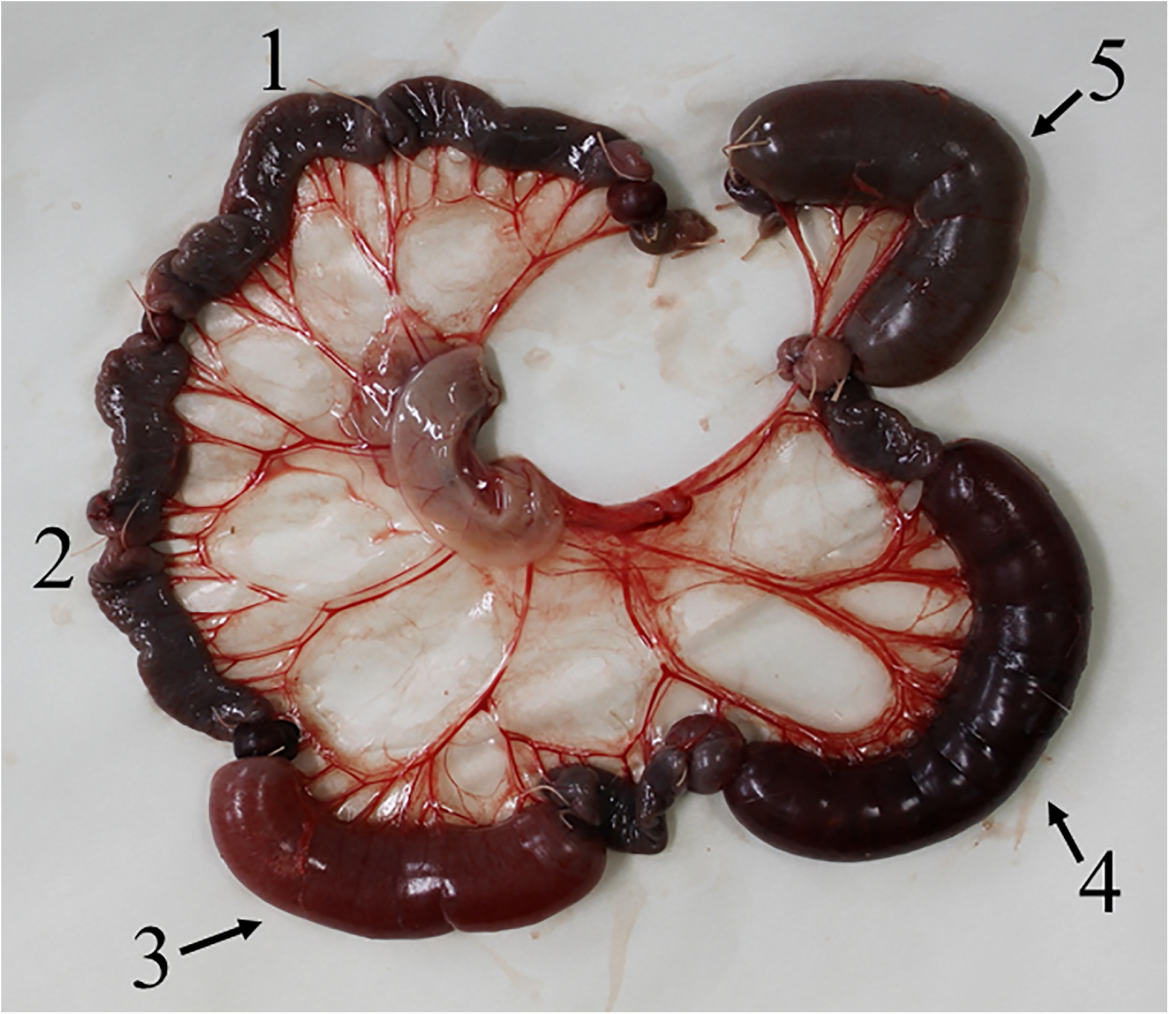
The rabbit ileum loop test for the rCPILE of *Clostridium perfringens* strain W5052. Fluid accumulation was observed in several ileal loops (arrows) in response to the injected rCPILE. Loop 1, saline; Loop 2, rCPIILE-a (0.1 μg) and trypsin-digested rCPILE-b (0.9 μg); Loop 3, rCPILE-a (1 μg) and trypsin-treated rCPILE-b (9 μg); Loop 4, rCPILE-a (10 μg) and trypsin-treated rCPILE-b (90 μg); and Loop 5, cholera toxin (1 μg).

The ileal loops injected with saline as a negative control (Loop No. 1 of Fig 13) and the loop injected with a mixture of 0.1 μg of rCPILE-a and 0.9 μg of the trypsin-treated rCPILE-b (Loop No. 2) did not show any pathological effects. The loops injected with cholera toxin (Loop No. 5) and a mixture of the rCPILE-a and trypsin-treated rCPILE-b (Loop No. 3, 1 μg and 9 μg and Loop No. 4, 10 μg and 90 μg) were swollen and became dark red, and fluid accumulation was observed in these loops. The F/A ratios of the loops injected with 1 μg of cholera toxin ranged from 0.5 to 1.3. The F/A ratios of the loops injected with a total 10 μg of CPILE ranged from 0.4 to 1.0 (Table 2). The fluid accumulation was neutralized by the sera mixture of anti-rCPILE-a and anti-rCPILE-b (data not shown). This finding indicated that CPILE exerted enterotoxic effects.

**Table 2:** The enterotoxic activity of the recombinant CPILE-a and CPILE-b of *Clostridium perfringens* strain W5052.

Sample	Rabbit							
	No. 1		No. 2		No. 3		No. 4	
	Volume(ml)/Length(cm)	F/A*	Volume(ml)/Length(cm)	F/A	Volume(ml)/Length(cm)	F/A	Volume(ml)/Length(cm)	F/A
Saline	0/5	0	0/4	0	0/4.6	0	0/5.4	0
CPILE-a** 0.1 μg + CPILE-b*** 0.9 μg	Not tested	—	0/7	0	0/5.1	0	0/5.5	0
CPILE-a 1.0 μg + CPILE-b 9.0 μg	2/4.5	0.4	8/8	1	5/5.8	0.9	8/7.8	1
CPILE-a 10 μg + CPILE-b 90 μg	4.5/6.6	0.7	7.5/7.4	1	10/9.9	1	12.5/9	1.4
Cholera toxin (1 μg)	2.5/5	0.5	7.5/7.4	1	12.5/13	1	10/7.8	1.3

*Fluid/Accumulation ratio (ml/cm)

** recombinant CPILE-a

*** recombinant CPILE-b.

### Production of CPILE of *C*. *perfringens* strain W5052 in the culture

**Fig 14:**
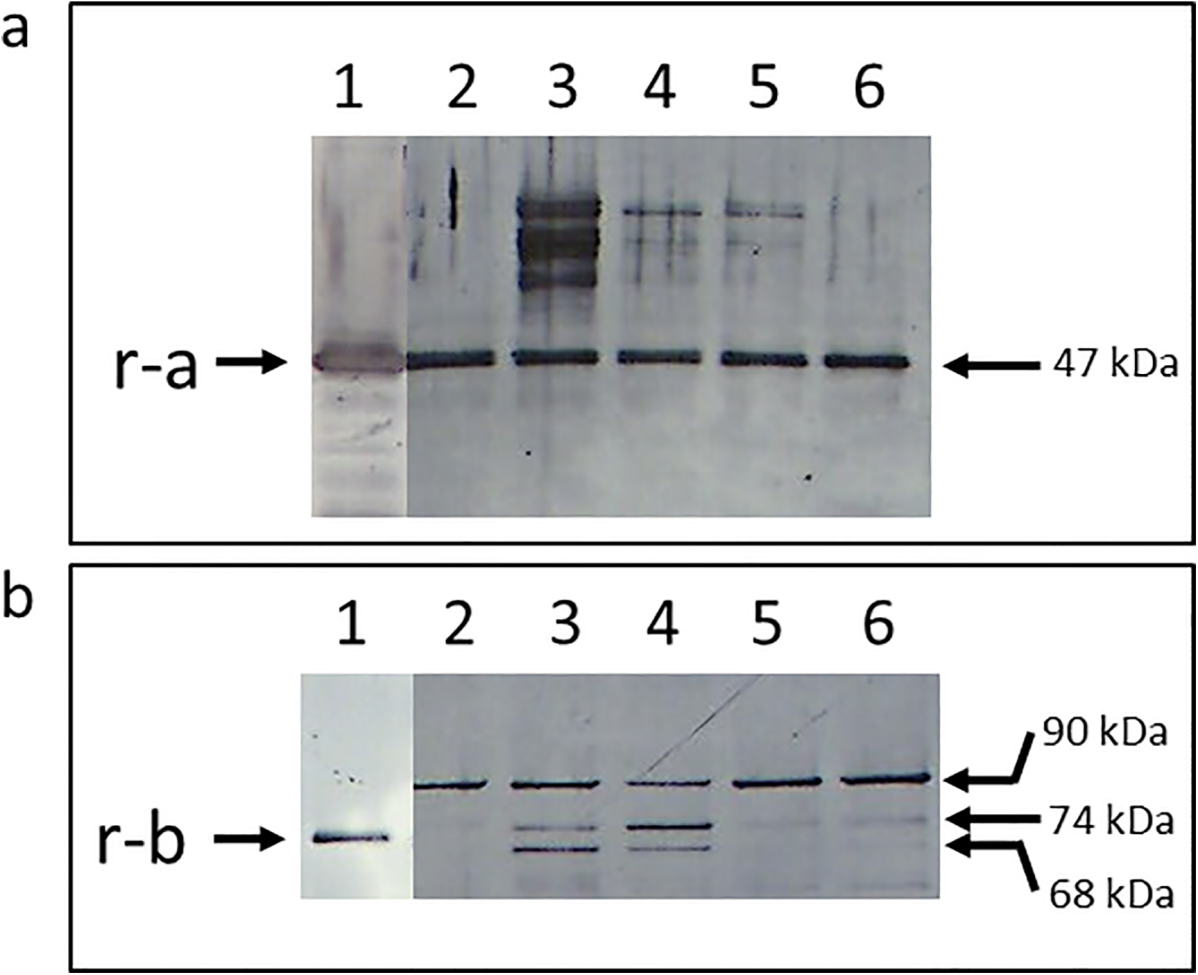
The immunoblotting analysis of the culture of *Clostridium perfringen*s strain W5052. *C*. *perfringens* strain W5052 strain was cultured in modified Duncan-Strong medium for the indicated periods. Aliquots of the culture were subjected to SDS-PAGE. The CPILE-a and -b were visualized by using the antibodies against the rCPILE-a (panel a) and trypsin-treated rCPILE-b (panel b). Lane 1, rCPILE-a or rCPILE-b as a positive control; Lane 2, the culture on day one; Lane 3, the culture on day two; Lane 4, the culture on day three; Lane 5, the culture on day five; and Lane 6, the culture on day 7. r-a indicates the position of recombinant CPILE-a, and r-b indicates recombinant CPILE-b.

A band with a molecular weight of 47 kDa that bound to the antibody against rCPILE-a was observed in the lane that migrated with the protein from a one-day culture of strain W5052, as well as the lane that migrated with the purified rCPILE-a (Fig 14, top). Although a couple of bands were observed in the upper positions, the 47 kDa band migrated at the same position in the sample from both two- and four-day cultures. The bands with a molecular weight of 90, 74, and 68 kDa were detected in the culture of W5052 using the antibody against the trypsin-treated rCPILE-b. The band of 74 kDa migrated at the same position as the trypsin-treated rCPILE-b (Fig 14, bottom). The protein with a molecular weight of 90 kDa was a putative intact CPILE-b. The protein with a molecular weight of 68 kDa was a degraded product of CPILE-b.

## Discussion

*C*. *perfringens* strain W5052 was isolated from stool samples of patients with food poisoning that was considered to have been caused by *C*. *perfringens* based on the epidemiological findings. However, strain W5052 did not harbor the enterotoxin gene nor produce the known enterotoxin, CPE. The culture supernatant of strain W5052 was positive in the ileal loop test, suggesting the presence of a new enterotoxin in the culture [20]. We herein describe the identification and characterization of the new enterotoxin and discussed its significance in the epidemiology of *C*. *perfringens* food poisoning.

The new candidate enterotoxin was identified by the next generation DNA sequencing of the genome of strain W5052 and a mass analysis of the partially purified enterotoxin. The genome of W5052 contained a gene encoding the homologue of *C*. *spiroforme* iota-like toxin. The mass spectrometry analysis revealed that a fraction of the partially purified toxin contained small peptides similar to components a and b of *C*. *perfringens* iota toxin. The gene encoding the candidate homologue was cloned and sequenced, and a recombinant protein of the candidate homologue was prepared, using the sequence obtained by next generation sequencing. The candidate homologue consisted of two components, a and b. The amino acid sequence of the component a homologue of W5052 was similar to that of iota-like toxin Sa of *C*. *spiroforme*, iota toxin ia of *C*. *perfringens*, C2 toxin component I of C. *botulinum*, and ADP-ribose transferase (cdtA) of *C*. *difficile* (77–83% similarity). The amino acid sequence of the component b homologue of W5052 was similar to that of component b of these toxins (77–81% similarity). The toxins described above, including the toxin of W5052 are binary toxins composed of both enzymatic and binding components [2, 3, 12, 28]. The new enterotoxin components a and b were named *C*. *perfringens* iota-like enterotoxin, CPILE-a and CPILE-b, respectively, according to the best homology.

The rCPILE-a possessed NADase and ADP-ribose transferase activity. The rCPILE-b treated with trypsin showed cytotoxic activity. The addition of rCPILE-a enhanced the cytotoxicity of the trypsin-treated rCPILE-b. The mixture of the rCPILE-a and trypsin-treated rCPILE-b evoked fluid accumulation in the ileal loop of rabbits, suggesting that the candidate had enterotoxic activity. All of the findings described herein demonstrated that the new enterotoxin of strain W5052 is a homologue of other binary toxins and that it is a member of the enterotoxin family.

When the amino acid sequence of CPILE-a was compared with that of the enzymatic component of the binary toxin family members, the arginine residue as the catalytic center and a Glu-X-Glu (EXE) motif were completely conserved, and a STS-motif had a similarity of 100%. When we compared the amino acid sequences of CPILE-b with that of the enzymatic component of a binary toxin family member, the aspartic acid residue cluster involved in the interaction of the enzymatic component in the presence of Ca^2+^ [27] was completely conserved. Based on a homology search, the binding component of the binary toxin members could be divided into four domains: binding to the enzymatic component, insertion into the membrane, oligomerization, and binding to the cell [16]. The amino acids, V578 and F799, of CPILE-b were estimated to represent the binding site to the cell receptor, but the amino acids of the iota toxin ib were not identical. A recent study reported that iota toxin ib bound to nonlipid rafts, formed an oligomer, and caused a rapid necrosis of A421 and A549 cells [19]. The trypsin-treated rCPILE-b alone showed cytotoxicity to L929 and Vero cells. However Vero cells were not susceptible to iota toxin ib [19], suggesting the presence of distinct receptors for the members of the iota toxin group. Lipolysis-stimulated lipoprotein receptor was identified as the receptor for *C*. *spiroforme* iota-like toxin [29]. The studies of the receptor for CPILE-b will be interesting.

The cytotoxicity of iota toxin was also facilitated when its ib component was treated with the λ-protease of *C*. *perfringens*, that is a zinc-dependent protease related to thermolysin [30]. Although proteolysis was necessary to activate the CPILE-b to induce its cytotoxicity, a homologue of λ-protease was not found in our database of the W5052 genome. The immunoblotting analysis of the culture of strain W5052 showed that the CPILE-b was cleaved. These findings indicate that another unknown endogenous protease(s) in strain W5052 would be responsible for the activation of CPILE-b.

The conserved signal peptide sequence region of CPILE-a and CPILE-b was not identified in the genome of strain W5052. This finding is in accordance with that of the C2 toxin of *C*. *botulinum*. The C2 toxin was produced during sporulation and not during vegetative growth [31]. CPILE was partially purified from the spore-forming medium cultured with strain W5052, but the expression of the mRNA for the *cpile-a* and *cpile-b* genes was detected in the cells cultured in the medium for vegetative growth. The mRNA expression and production of CPILE-a and -b must be examined in the further experiments to understand how and when *cpile-a* and *-b* are expressed and their proteins are synthesized.

Incidences of *C*. *perfringens* type A food-poisoning are currently confirmed by examinations to detect the gene and protein of the known enterotoxin, CPE. All of the isolates from the four incidences of *C*. *perfringens*-food poisoning expressed the mRNA of *cpile-a* and *-b* (Fig 5). Strain W5052, which was the particular strain examined in this study, produced CPILE-a and -b proteins in a modified DS medium (Fig 14). As presented in this communication CPILE possesses enterotoxic activity (Fig 13).

An incidence of food poisoning caused by a strain producing CPILE would be classified as *C*. *perfringens* food poisoning. However the isolates of the four incidences in which CPE was not produced and did not harbor the *cpe* gene, or express mRNA and protein of CPE, must be excluded from historic *C*. *perfringens* type A food poisoning. The coding sequence of *cpile-a* and *-b* is easy to amplify by the use of a general PCR technique (data not shown) and the antibody against CPILE-a and -b is currently available. It is epidemiologically convinced that the particular four incidences of food poisoning were caused by *C*. *perfringens* producing CPILE [20]. Although the data presented in this communication do not fully support that CPILE-producing strains are responsible for *C*. *perfringens* food poisoning, the goal would be closed. The presence of CPE protein in the patients’ feces is one of the important findings for conclusive diagnosis of *C*. *perfringens* type A food poisoning. The CPE can be detected by a reversed-passive latex agglutination (RPLA) test, that uses the antibodies against CPE. In a preliminary trial, we adapted the antibodies against the trypsin-treated rCPILE-b to the RPLA system and tried to detect the CPILE-b protein in the patients’ feces. The precise results will be reported soon in another opportunity.

*C*. *perfringens* type E produces iota toxin and alpha toxin. Iota toxin causes enteritis and sudden death in beef calves and lambs [32] but not in humans. Strain W5052 and other strains that originated from the particular incidences, harbor CPILE instead of iota toxin. Furthermore these strains cause diarrhea in human due to the enterotoxic action of CPILE. Based on these findings, new sub-classifications of *C*. *perfringens* could be proposed, namely types E1 and E2. Type E1 *C*. *perfringens*, which is pathogenic to animals, produces alpha and iota toxins; while type E2 *C*. *perfringens*, which is pathogenic to human, produces alpha toxin and CPILE. Strain W5052 belongs to type E2.

The gene of iota toxin of *C*. *perfringens* and of iota-like toxin of *C*. *spiroforme* is encoded in its plasmid and genome, respectively [33]. BEC, binary enterotoxin of *C*. *perfringens*, which was previously reported by Yonogi et al. [34], consists of two components: BECa and BECb. BEC showed higher similarity to iota toxin and iota-like toxin. The genes of BECa and BECb are located in a 54,5 kb plasmid. CPILE is also encoded in a plasmid (unpublished data). The results of a BLAST search indicated that the sequence of *cpile-a* and *cpile-b* perfectly matched that of *beca* and *becb*, respectively. This observation indicates that CPILE islikely BEC.

The mechanism by which genes of the iota toxin group move among Clostridia and by which genetic mutations raise is highly interesting. Our findings will facilitate the microbiological, epidemiological, and toxicological research on *C*. *perfringens*.
